# Increased expression of the microRNA 106b~25 cluster and its host gene MCM7 in corticotroph pituitary adenomas is associated with tumor invasion and Crooke’s cell morphology

**DOI:** 10.1007/s11102-017-0805-y

**Published:** 2017-04-21

**Authors:** Filip Garbicz, Dawid Mehlich, Beata Rak, Emir Sajjad, Maria Maksymowicz, Wiktor Paskal, Grzegorz Zieliński, Paweł K. Włodarski

**Affiliations:** 1 Laboratory of Centre for Preclinical Research, Department of Histology and Embryology, Medical University of Warsaw, Banacha 1B, 02-091 Warsaw, Poland; 2 Postgraduate School of Molecular Medicine, Warsaw, Poland; 3 Department of Neurosurgery, Military Institute of Medicine, Warsaw, Poland; 4 Department of Pathology and Laboratory Diagnostics, M. Skłodowska-Curie Memorial Cancer Centre and Institute of Oncology, Warsaw, Poland; 5 Department of Internal Diseases and Endocrinology, Public Central Teaching Hospital Medical University of Warsaw, Warsaw, Poland

**Keywords:** Pituitary adenoma, ACTHoma, Crooke’s cell adenoma, MicroRNA, MiR-106b~25 cluster, MCM7

## Abstract

**Purpose:**

MCM7 (minichromosome maintenance complex component 7), a DNA replication licensing factor, is a host gene for the oncogenic miR-106b~25 cluster. It has been recently revealed as a relevant prognostic biomarker in a variety of cancers, including pituitary adenomas. The purpose of this study was to assess whether miR-106b~25 and MCM7 levels correlate with tumor invasiveness in a cohort of ACTH-immunopositive adenomas.

**Methods:**

Tissue samples were obtained intraoperatively from 25 patients with pituitary adenoma. Tumor invasiveness was assessed according to the Knosp grading scale. MCM7, Ki-67 and TP53 levels were assessed by immunohistochemical staining, while the expression of miR-106b-5p, miR-93-5p, miR-93-3p and miR-25-3p were measured using quantitative real-time PCR performed on RNA isolated from FFPE tissues.

**Results:**

We have found a significant increase in MCM7 and Ki-67 labeling indices in invasive ACTHomas. Moreover, MCM7 was ubiquitously overexpressed in Crooke’s cell adenomas. The expression of miR-93-5p was significantly elevated in invasive compared to noninvasive tumors. In addition, all four microRNAs from the miR-106b~25 cluster displayed marked upregulation in Crooke’s cell adenomas. Remarkably, MCM7 and miR-106b-5p both strongly correlated with Knosp grade. A combination of MCM7 LI and miR-106b~25 cluster expression was able to accurately differentiate invasive from noninvasive tumors and had a significant discriminatory ability to predict postoperative tumor recurrence/progression.

**Conclusions:**

miR-106b~25 and its host gene MCM7 are potential novel biomarkers for invasive ACTH-immunopositive pituitary adenomas. Additionally, they are both significantly upregulated in rare Crooke’s cell adenomas and might therefore contribute to their aggressive phenotype.

## Introduction

**Fig. 1:**
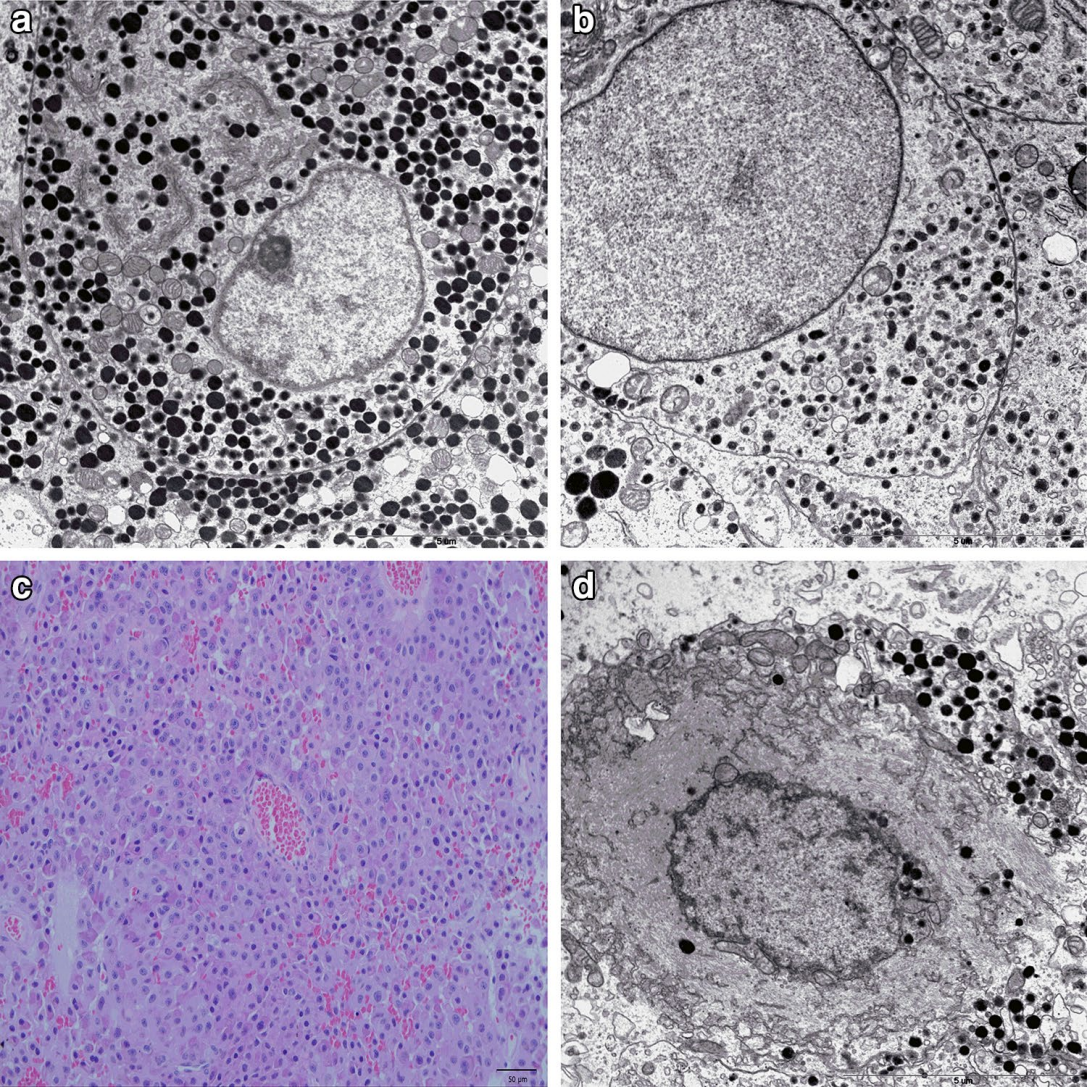
Pituitary corticotroph adenomas. **a** Ultrastructural features of densely granulated corticotroph adenoma: well-developed organelles, numerous, variable in shape, and electron dense secretory granules. Original magnification ×7400. **b** Electron microscopy of sparsely granulated corticotroph adenoma: small and scanty secretory granules. Original magnification ×9700. **c** H&E staining of Crooke’s cell adenoma (×200). **d** Ultrastructural features of Crooke’s cell adenoma: excessive accumulation of perinuclear cytokeratin filaments. Original magnification ×9700

Corticotroph adenomas represent approximately 5–10% of all pituitary adenomas (PAs) [1, 2]. Their most common clinical manifestation is Cushing’s disease that is caused by hormonally active, adrenocorticotropin (ACTH) secreting tumors. ACTH-producing pituitary adenomas are the most common non-iatrogenic cause of Cushing’s syndrome (hypercortisolemia from any source) and account for an estimated 70% of all cases [3, 4]. In 85% of the cases of Cushing’s disease, the tumor is a well-demarcated microadenoma [5]. A minority of corticotroph adenomas (7–9%) are hormonally silent and usually present as macroadenomas causing mass effect, including headache and vision defects [6]. When compared to other types of silent adenomas (mainly null cell and gonadotroph adenomas), silent corticotroph adenomas (SCAs) are considered to recur earlier and more frequently [7–9]. Based on their morphology observed using electron microscopy and low molecular weight keratin (LMWK)-CAM5.2 immunohistochemical staining, corticotroph adenomas are divided into densely (DG-ACTH) and sparsely (SG-ACTH) granulated that correspond to subtype I and subtype II silent corticotroph adenomas, respectively [10] (Fig. 1a, b). Another variant of corticotroph pituitary adenomas is Crooke’s cell adenoma (CCA). Crooke’s cells are corticotrophs that, in the setting of glucocorticoid excess (regardless of its etiology), undergo massive accumulation of perinuclear cytokeratin filaments, giving their cytoplasm a distinct hyalinized appearance on hematoxylin and eosin stain (Fig. 1c, d). The hyaline change is assumed to represent a response of normal, non-neoplastic corticotroph cells to elevated glucocorticoids level and is generally not observed in adenoma cells except Crooke’s cell adenoma [6, 11, 12]. This type of adenoma is considered to be innately aggressive, invasive, associated with significant morbidity and is prone to recurrence rates as high as 60% [12, 13].

Predicting the behavior of pituitary adenomas in terms of invasiveness, aggressiveness, recurrence and postoperative patient outcome requires tumor markers that correlate accurately with the mentioned features. The 2004 WHO classification of endocrine tumors besides typical benign pituitary adenomas and pituitary carcinomas distinguishes a group of tumors exhibiting ‘borderline or uncertain behavior’ classified as atypical adenomas [14, 15]. Atypical adenomas are characterized by elevated mitotic index, Ki-67 labeling index (LI) > 3% and extensive nuclear reactivity for p53 [15]. In addition, the expression of MGMT (*O*-6-methylguanine-DNA methyltransferase) can be assessed in order to predict PA’s sensitivity to temozolomide, an alkylating chemotherapeutic [13].

Despite their widespread use and recommendation by 2004 WHO classification, the usefulness of Ki-67 index and p53 immunopositivity as reliable markers of pituitary tumor biology remains controversial. Some authors suggest that these markers have no superiority over the mentioned detailed histological typing of pituitary adenomas [16]. Other biomarkers of PA aggressiveness include fibroblast growth factor receptor 4 (FGFR 4), matrix metalloproteinases (MMPs), presence of growth hormone receptor mutations, loss of chromosome arm 11p and/or 11q and pituitary tumor transforming gene 1 (PTTG1) overexpression [17]. Most recently, Coli et al. have reported a significantly increased expression of minichromosome maintenance complex component 7 (MCM7) in invasive ACTH-producing PAs [18] and proposed MCM7 labeling index as a prognostic marker of clinical outcome in PA patients.

**Fig. 2:**
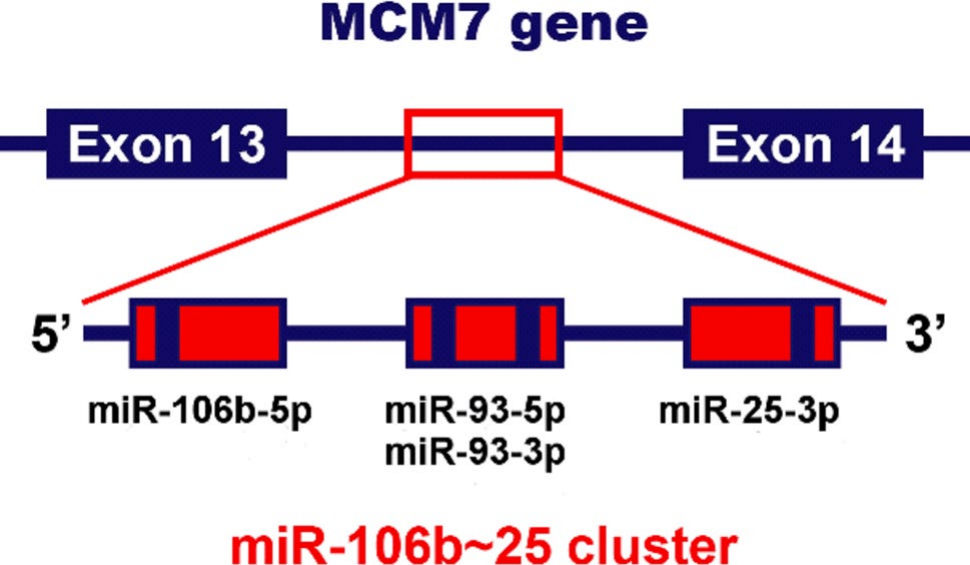
A schematic representation of the polycistronic miR-106b~25 cluster and its host gene MCM7, located on chromosome 7q22

MicroRNAs (miRNAs) are known to play a crucial role in the pathogenesis of cancer and recently they are emerging as novel neoplastic biomarkers [19]. They are short (18–24 nucleotides in length), noncoding RNA molecules that regulate gene expression at the post-transcriptional level. MiRNAs influence mRNA stability and translation by binding to regulatory sites located primarily in the 3′ untranslated region (UTR) of the targeted transcripts [20, 21]. Recent studies have shown that miR-106b~25 cluster, consisting of highly conserved miRNAs: miR-25, miR-93 and miR-106b, is overexpressed in numerous types of cancer including gastric cancer, hepatocellular carcinoma, esophageal adenocarcinoma, neuroblastoma, and prostate cancer [22–27]. Interestingly, it is located within the 13th intron of MCM7 (Fig. 2), an oncogene acting in cooperation with the aforementioned cluster in promoting cancer progression [27].

The aim of this study was to investigate the expression pattern of miR-106b~25 cluster and MCM7 in corticotroph adenomas, including CCAs, with regard to their histopathological subtype, other markers of invasiveness, and clinical behavior.

## Materials and methods

### Patient samples

A retrospective group of 25 patients diagnosed with Cushing’s disease and ACTH-immunopositive pituitary adenoma were enrolled for this study. Tumor samples were retrieved from patients undergoing planned surgery at the Department of Neurosurgery in Military Institute of Medicine in Warsaw. PAs were resected from 5 male and 20 female patients, with a mean age of 47.7 (range 17–78 years) at the time of diagnosis. The detailed clinicopathological characteristics of all patients included in this study are presented in Table 1. Two samples from patients no. three and seven were included: one from a primary and one from a recurrent tumor. All slides were examined by a board-certified pathologist. Tumors were classified according to their size, determined by magnetic resonance imaging (MRI), as microadenomas (<10 mm) or macroadenomas (>10 mm). The diagnosis of invasive PAs was based on preoperative MRI assessed using Knosp grading scale combined with intraoperative evaluation [28, 29]. The diagnosis of CCA, SG-ACTH and DG-ACTH was based on examination of PAs using light and electron microscopy. After surgery, patients were followed-up through MRI and biochemical tests, receiving appropriate medical intervention, including additional surgery or drug therapy in case of tumor recurrence or progression. The follow-up results were defined according to the criteria used by Righi et al. [30]. Recurrence was defined as radiological evidence of disease relapse in patients without residual tumor after surgical therapy, and progression was defined as tumor regrowth based on MRI and/or evidence of increased plasma hormone levels in patients with residual tumor. A local ethics committee approved all aspects of this study in accordance with the Helsinki Declaration.

**Table 1:** The clinicopathological characteristics of the patients included in this study

Sample ID	Sex	Age	Tumor type	KNOSP grade	Tumor dimension	Immunohistochemistry (% of stained cells)				Detailed pathological description
						TP53	MIB1	MGMT	MCM7	
1	F	33	Crooke’s cell adenoma	0	Microadenoma	1	3	75	20 focally: 50	Crooke‘s cell variant, atypical
2	M	49	Crooke’s cell adenoma	4	Macroadenoma	1	1	0	50	Crooke‘s cell variant
3a	F	55	Crooke’s cell adenoma	4	Macroadenoma	3	3	0	30	Crooke‘s cell variant, atypical
3b	F	56	Crooke’s cell adenoma	4	Macroadenoma	10	10	10	40	Crooke‘s cell variant, atypical
4	F	33	Crooke’s cell adenoma	0	Microadenoma	3	3	90	20 focally: 80	Crooke’s cell variant, atypical
5	F	74	Invasive PA	3	Macroadenoma	1	3	90	20	SG-ACTH, atypical, single mitotic figures
6	F	48	Invasive PA	3	Macroadenoma	0	3	N/A	10	DG-ACTH
7a	M	55	Invasive PA	3	Macroadenoma	N/A	N/A	N/A	N/A	SG-ACTH
7b	M	55	Invasive PA	3	Macroadenoma	50	20	0	30	SG-ACTH, atypical
8	F	59	Invasive PA	2	Macroadenoma	0	3	0	10	SG-ACTH
9	F	36	Invasive PA	4	Macroadenoma	2	20	70	40 focally: 60	DG-ACTH, atypical
10	F	66	Invasive PA	4	Macroadenoma	2	5	50	50	SG-ACTH, atypical
11	M	61	Invasive PA	4	Macroadenoma	25	5	20	30	SG-ACTH, atypical, nuclear atypia
12	F	17	Invasive PA	3	Macroadenoma	3	3	90	10	SG-ACTH, high nuclear atypia
13	F	78	Invasive PA	4	Macroadenoma	1	1	90	5 focally: 30	DG-ACTH
14	F	58	Invasive PA	2	Macroadenoma	0	3	100	50	DG-ACTH, atypical
15	F	43	Invasive PA	2	Macroadenoma	1	3	100	20	DG-ACTH, atypical
16	F	44	Invasive PA	2	Microadenoma	1	3	30	15	DG-ACTH
17	F	58	Noninvasive PA	1	Macroadenoma	1 focally: 20	1	90	1 focally: 20	–
18	M	49	Noninvasive PA	0	Microadenoma	0	1	50	5	DG-ACTH
19	F	21	Noninvasive PA	0	Microadenoma	1	3	0	5	–
20	F	51	Noninvasive PA	0	Microadenoma	1	1	90	1	SG-ACTH, alpha(+/−)
21	F	42	Noninvasive PA	0	Microadenoma	1	1	70	20 focally: 80	DG-ACTH
22	F	36	Noninvasive PA	0	Microadenoma	0	1	50	1	DG-ACTH
23	F	44	Noninvasive PA	1	Microadenoma	1	1	100	3	DG-ACTH
24	F	34	Noninvasive PA	0	Microadenoma	20	3	90	N/A	DG-ACTH, atypical
25	M	34	Noninvasive PA	1	Macroadenoma	2	3	50	30	DG-ACTH

### Immunohistochemistry (IHC)

Tissue specimens were fixed in 10% buffered formalin, embedded in paraffin and routinely stained with hematoxylin and eosin. Immunohistochemical staining was performed on paraffin-embedded sections according to the labeled EnVision Flex Visualization System (Dako) with DAB (3,3′-diaminobenzidine) as chromogen, using antibodies against: growth hormone (GH, dilution 1:500), prolactin (PRL, 1:200), ACTH (1:500), β-TSH (1:200), β-FSH (1:500), β-LH (1:500), MGMT (MT 23.2 clone; 1:100)—all antibodies from Thermo Fisher Scientific; the glycoprotein α-subunit (dilution 1:100) from Bio-Rad, UK; TP53 and Ki-67 (MIB-1 clone, ready to use) from Dako, and MCM7 (141.2 clone; 1:200) from Santa Cruz Biotechnology.

### Electron microscopy

Small pieces of each tumor were fixed in 2.5% glutaraldehyde, postfixed in 1% osmium tetroxide, dehydrated and embedded in Epon. Ultrathin sections counterstained with uranyl acetate and lead citrate were examined with a Philips CM120 BioTWIN electron microscope.

### RNA isolation

Total RNA was isolated from FFPE (formalin-fixed paraffin-embedded) tissue blocks as previously described [31] using the RecoverALL Total Nucleic Acid Isolation Kit for FFPE (Thermo Fisher Scientific). Approximately 15 mg of unsectioned core samples were cut out from the paraffin block and crushed on dry ice. Subsequently, samples were deparaffinized using a series of xylene and ethanol washes, digested with protease and DNase, and eluted with 60 µl of Elution Solution. The quantity and purity of isolated RNA were assessed by the absorbance measurements at wavelengths of 260 and 280 nm on NanoDrop 2000 spectrophotometer (Thermo Fisher Scientific) using 1 µl of RNA. We assumed that samples with OD 260/280 ratios between 1.8 and 2.1 were acceptable for further analysis.

### Reverse transcription (RT) and quantitative polymerase chain reaction (qPCR)

MicroRNA expression levels were determined using TaqMan Advanced miRNA Assays (Thermo Fisher Scientific) according to the manufacturer’s protocol. The following miRNA assays were used: hsa-miR-25-3p (assay ID: 477994), hsa-miR-93-3p (assay ID: 478209), hsa-miR-93-5p (assay ID: 478210) and hsa-miR-106b-5p (assay ID: 478412). 2–10 ng of RNA isolated from each FFPE sample was reverse transcribed using TaqMan Advanced miRNA cDNA Synthesis Kit (Thermo Fisher Scientific). The subsequent qPCR reactions were performed in a total volume of 10 µl. Each qPCR reaction consisted of: 0.5 µl of TaqMan miRNA Advanced Assay, 5 µl of TaqMan Fast Advanced Mastermix, 2 µl of H_2_O and 2.5 µl of cDNA (diluted 1:10 with H_2_O). Expression levels of U6 snRNA (Assay ID: 001973) were determined using TaqMan MicroRNA Assay (Thermo Fisher Scientific). Reverse transcription reactions in a total volume of 15 µl were performed with miRNA-specific stem-loop RT primer using TaqMan MicroRNA Reverse Transcription Kit (Thermo Fisher Scientific), followed by qPCR reactions in a total volume of 10 µl. Each qPCR reaction consisted of 0.5 µl of U6 TaqMan Assay, 5 µl of TaqMan Universal PCR Master Mix II no UNG (Thermo Fisher Scientific), 0.67 µl of cDNA (diluted 1:10 with H_2_O) and 3.83 µl of H_2_O.

All qPCRs were performed in MicroAmp Fast Optical 96 Well Reaction Plates (Thermo Fisher Scientific) using Applied Biosystems 7500 Fast Real-Time PCR System with 7500 Software V2.0.6 (Thermo Fisher Scientific). Samples were assayed in triplicates, and the obtained CT values for target genes and housekeeping control (U6) were used to calculate relative gene expression using the 2^−ΔCT^ method as described previously [32].

### Statistical analyses

Mann–Whitney and Kruskal–Wallis tests were applied to assess mean differences between groups. To determine the statistical dependence between miRNA expression and clinicopathological characteristics Spearman’s or Pearson correlation coefficients were used when appropriate. The area under the receiver operating characteristic curve (AUC) was used to assess the sensitivity and specificity of the biomarkers. Logistic regression was applied to obtain a combined biomarker expression score. Logistic regression and ROC curve analysis were performed in SPSS 18.0 software (SPSS Inc.). All the other statistical tests were performed using GraphPad Prism 6 (GraphPad Software Inc.). All values are presented as mean ± SEM unless indicated otherwise. A p-value of <0.05 was considered statistically significant.

## Results

### Invasive ACTHomas exhibit elevated Ki-67 and MCM7 labeling indices

**Fig. 3:**
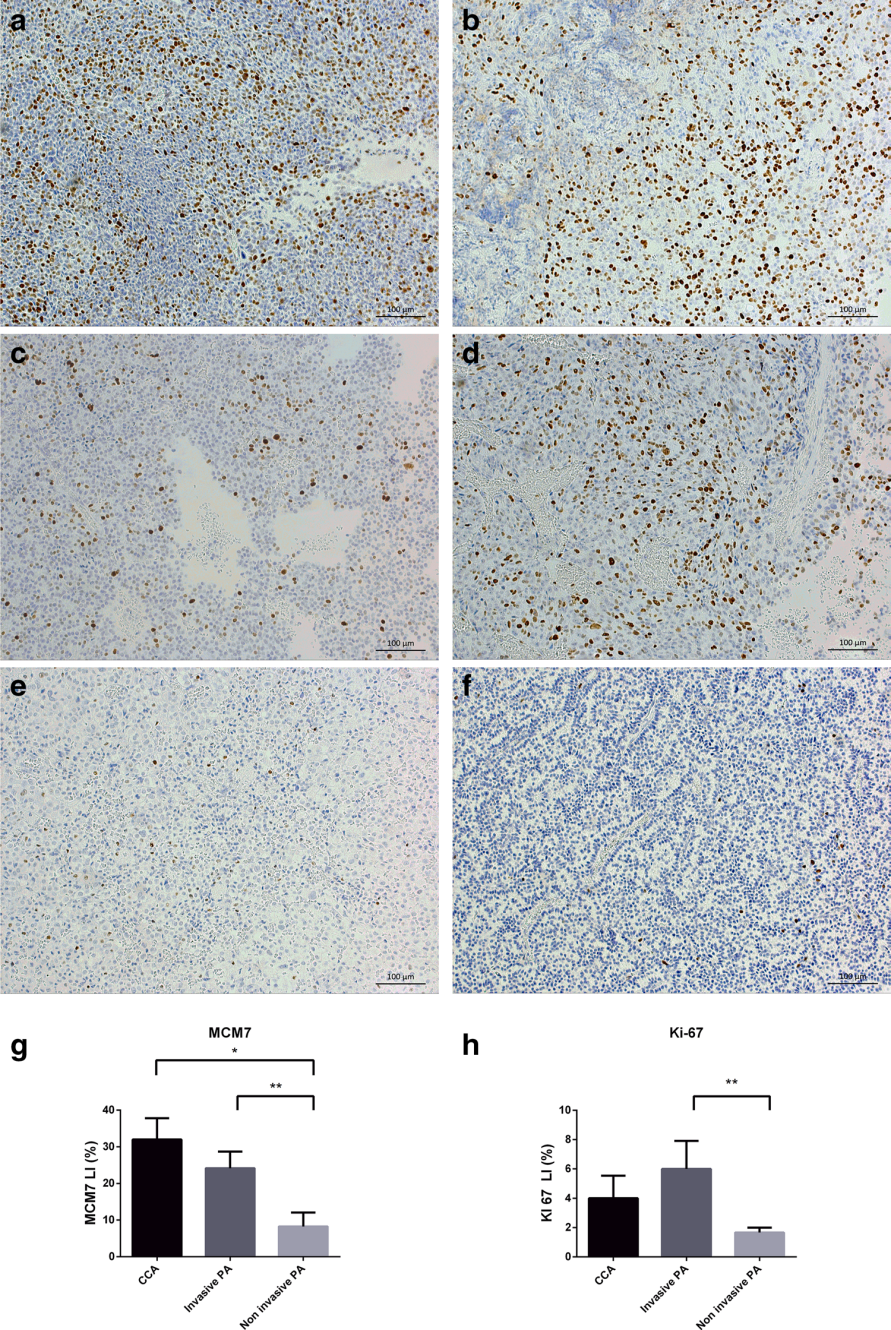
Immunohistochemical analysis of MCM7 expression in ACTH-producing pituitary adenoma samples. Presented are representative cases of MCM7-stained Crooke’s cell (**a**—2, **b**—3b), invasive (**c**—6, **d**—7a) and noninvasive (**e**—20, **f**—22) PAs. MCM7 (**g**) and Ki-67 (**h**) LIs are presented as % of positively stained cells in examined PAs. Columns represent means ± SEM. The subgroups were compared using the two-sided Mann–Whitney test: *p < 0.05, **p < 0.01, ***p < 0.001

We evaluated the expression of Ki-67, MCM7, TP53 and MGMT and sought associations with the clinicopathological characteristics of PA patients: age, tumor size, tumor invasiveness and the histological variant of the tumor (Table 2). The overall mean LIs (%) were: 20.64 ± 3.23 (MCM7), 4.12 ± 0.99 (Ki-67), 5.04 ± 2.15 (TP53), 56.2 ± 7.59 (MGMT). Ki-67 LI correlated significantly with MCM7 LI (Pearson r = 0.4316, p = 0.0312) and TP53 LI (Pearson r = 0.6144, p = 0.0008). No other marker pairs detected by IHC showed a significant correlation between them. Macroadenomas exhibited a higher LI of MCM7 (p = 0.0102) and Ki-67 (p = 0.0435) than microadenomas. Both markers were also more abundantly expressed in invasive PAs comparing to noninvasive tumors (p = 0.0065 and p = 0.0098, respectively). MCM7 LI and Ki-67 LI were similar in CCAs and non-Crooke invasive PAs, albeit significantly higher than in noninvasive tumors (Fig. 3). Neither MGMT LI nor TP53 LI showed any correlation with the clinicopathological data considered.

**Table 2:** Immunohistochemical staining of TP53, Ki-67, MGMT and MCM7 according to the clinicopathological characteristics of the patients

Variables	n	TP53 LI (%)	P	Ki-67 LI (%)	P	MGMT LI (%)	P	MCM7 LI (%)	P
Age									
<50	15	2.47 ± 1.28	0.4548	3.47 ± 1.21	0.2856	61.79 ± 8.98	0.5755	17.79 ± 3.82	0.4225
>50	12	8.55 ± 4.71		5.0 ± 1.69		49.09 ± 13.10		24.27 ± 5.52	
Tumor dimension									
Macroadenoma	17	6.38 ± 2.9	0.3411	5.438 ± 1.52	0.0435	50.67 ± 10.78	0.5325	26.63 ± 4.13	0.0102
Microadenoma	10	3.3 ± 1.92		2.0 ± 0.33		64.50 ± 10.07		10.0 ± 2.85	
Invasiveness of the tumor									
Invasive PA	16	6.67 ± 3.51	0.4862	5.73 ± 1.59	0.0098	46.43 ± 11.22	0.2755	27.33 ± 4.11	0.0065
Noninvasive PA	11	2.82 ± 1.74		1.91 ± 0.31		68.64 ± 8.84		10.60 ± 3.40	
Tumor subtype									
CCA	5	3.6 ± 1.66	0.1250	4.00 ± 1.55	0.2343	35.00 ± 19.62	0.1578	32.00 ± 5.83	0.2228
DG-ACTH	12	2.42 ± 1.61		3.58 ± 1.52		72.73 ± 7.52		18.09 ± 4.85	
SG-ACTH	8	11.71 ± 7.2		5.71 ± 2.44		48.57 ± 15.95		21.57 ± 6.26	

Data is presented as % of stained cells (mean ± SEM). P values were calculated using the Kruskal–Wallis or Mann–Whitney tests

### MiR-106b~25 is upregulated in invasive ACTH-producing PAs

**Fig. 4:**
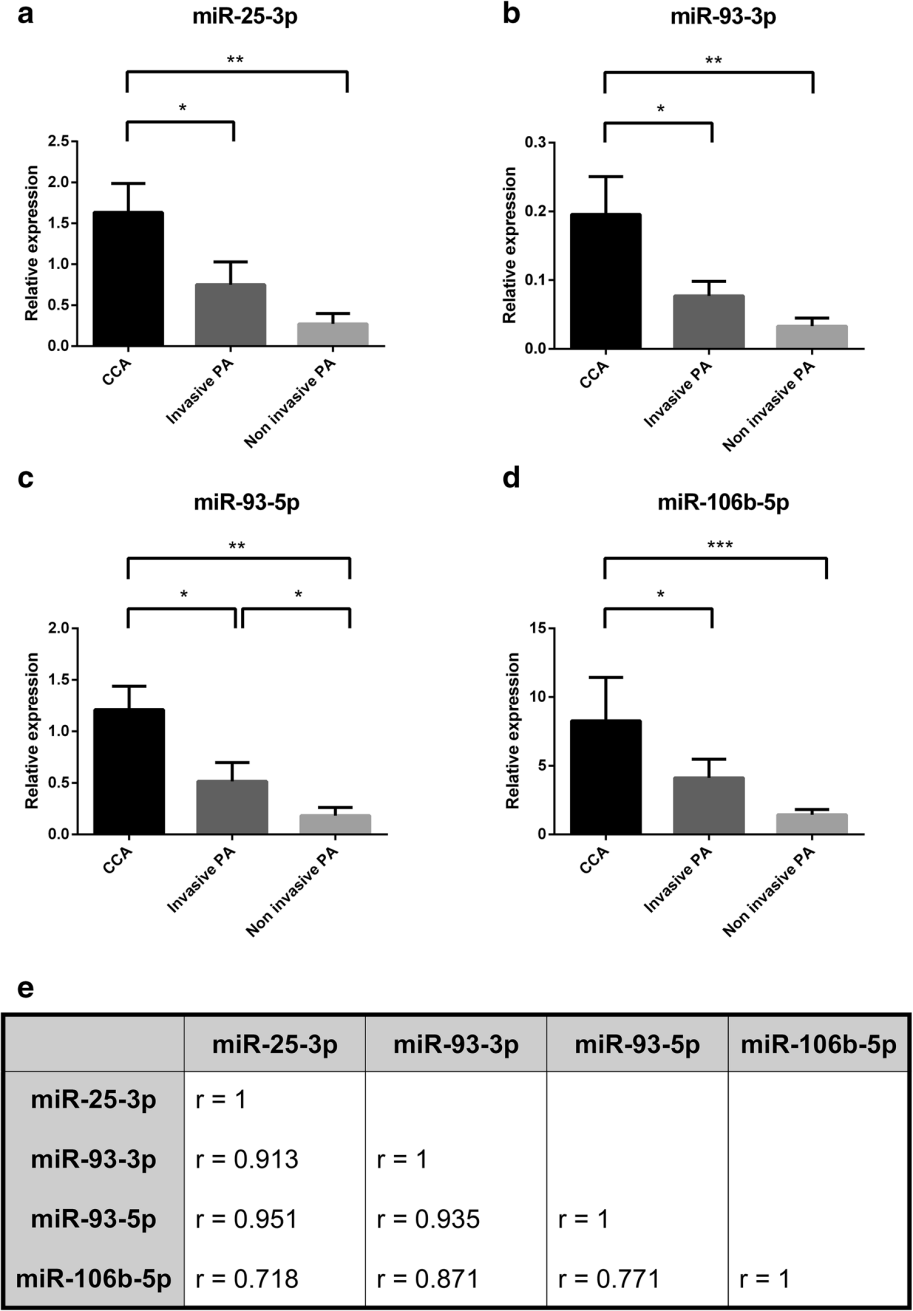
The elements of the miR-106b~25 cluster are coexpressed and upregulated in Crooke’s cell and invasive adenomas. **a**–**d** The expression of miR-106b~25 cluster elements in three subgroups of PAs was compared using the two-sided Mann–Whitney test: *p < 0.05, **p < 0.01, ***p < 0.001. **e** The table presents Pearson correlation coefficients between the expression levels of microRNAs studied. All miRNAs are significantly coexpressed in PAs (p < 0.0001 for all coefficients)

We analyzed the expression pattern of four miRNAs belonging to the miR-106b~25 cluster in invasive and noninvasive PAs. Since the most prominent changes were observed in CCAs, we separated them into a third subgroup for the following statistical analysis. The expression levels of all four miRNAs were significantly higher in CCAs than in invasive PAs (Fig. 4a–d). Although all miRNAs showed a general tendency towards a higher expression in invasive in comparison to noninvasive PAs, only the difference in miR-93-5p levels reached statistical significance. We also observed a strong reciprocal correlation between the expression of all miRNAs from the miR-106b~25 cluster (Fig. 4e).

### MCM7 LI correlates with the expression of miR-106b~25 and tumor’s Knosp grade

**Fig. 5:**
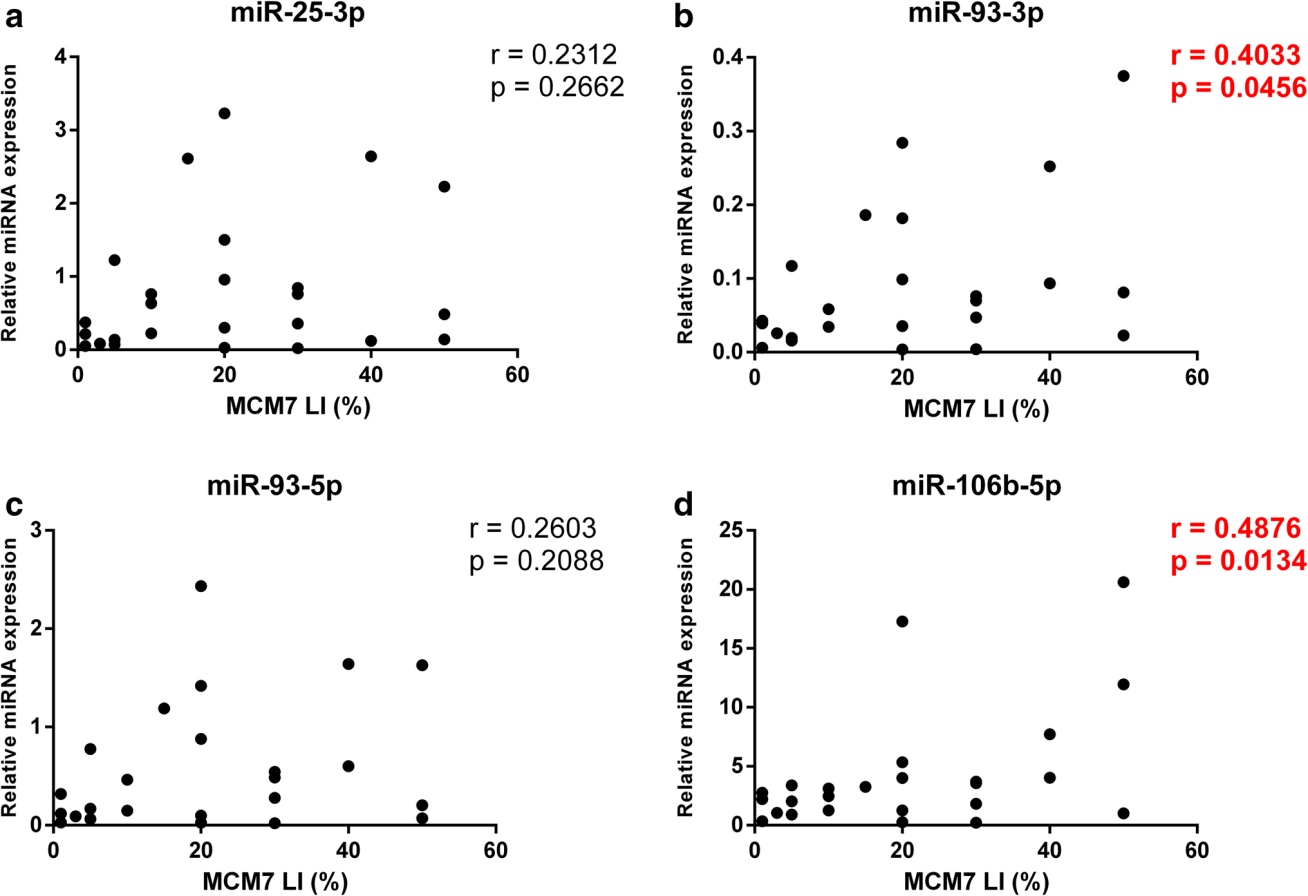
MCM7 and miR-106b-5p are coexpressed in ACTHomas. **a**–**d** The correlation was calculated using Pearson’s coefficient

**Fig. 6:**
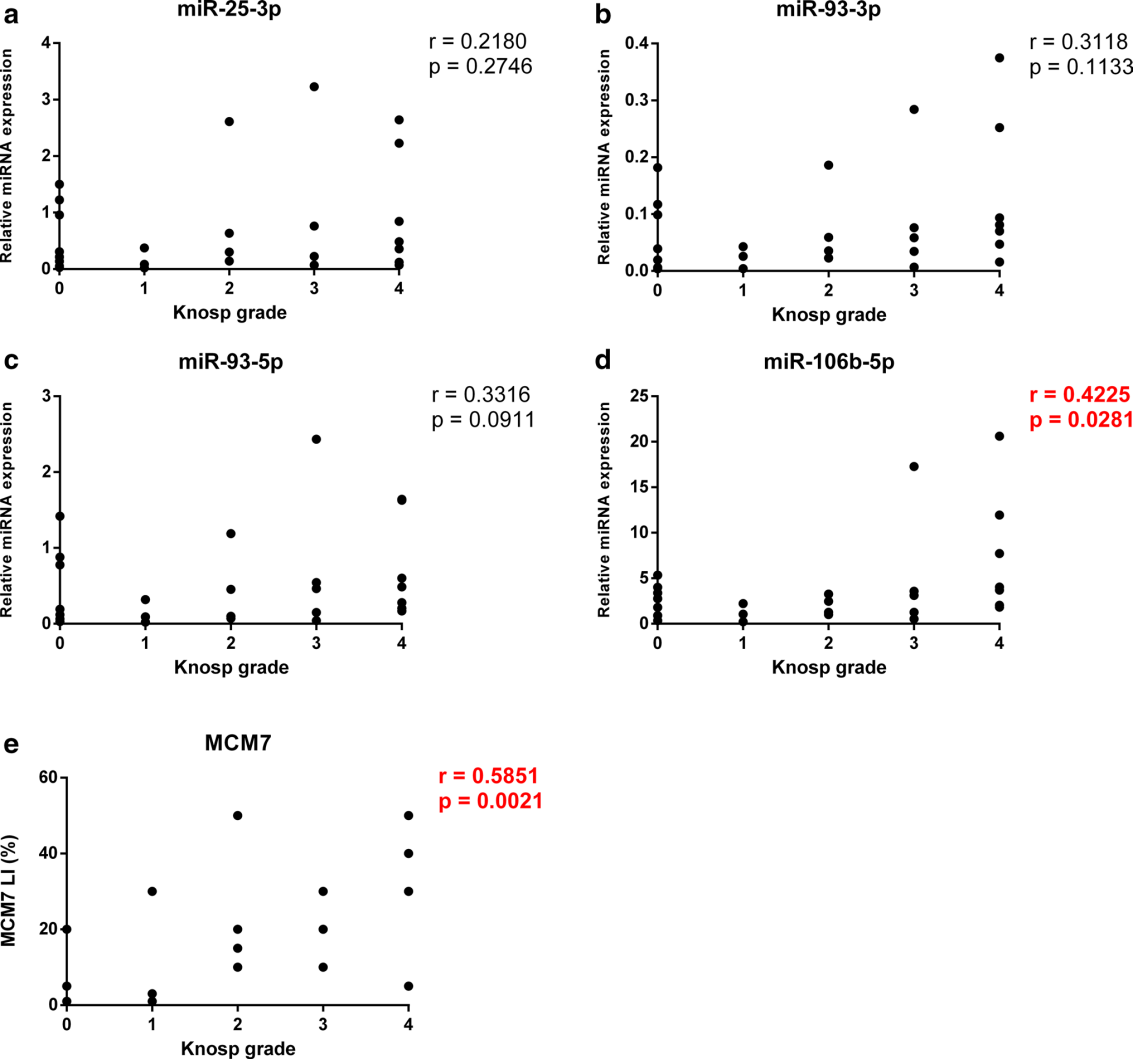
The expression of miR-106b-5p and MCM7 LI correlates with tumor’s Knosp grade. *Scatter plots* showing the relationship between tumor’s Knosp grade and miR-106b~25 expression (**a**–**d**) as well as MCM7 LI (**e**). Correlation was characterized by calculating Spearman’s rank coefficient

Since both miR-106b~25 and MCM7 share the same genomic location and were found to be overexpressed in invasive PAs, we analyzed the relationship between MCM7 LI and individual miRNA expression levels (Fig. 5). Among the four tested pairs, miR-106b-5p and miR-93-3p levels displayed a significant positive correlation with MCM7 LI (r = 0.4875, p = 0.0135 and r = 0.4033, p = 0.0456, respectively). Interestingly, Spearman’s test revealed that the invasiveness of the tumors measured using Knosp grading scale correlates with MCM7 LI as well as miR-106b-5p levels (r = 0.5851, p = 0.0021 and r = 0.4225, p = 0.0281, respectively). The rest of miRNAs from the miR-106b-25~cluster exhibited similar trends, however they did not reach statistical significance (Fig. 6).

### Combined MCM7 LI and miR-106b~25 expression differentiates invasive from noninvasive tumors

**Fig. 7:**
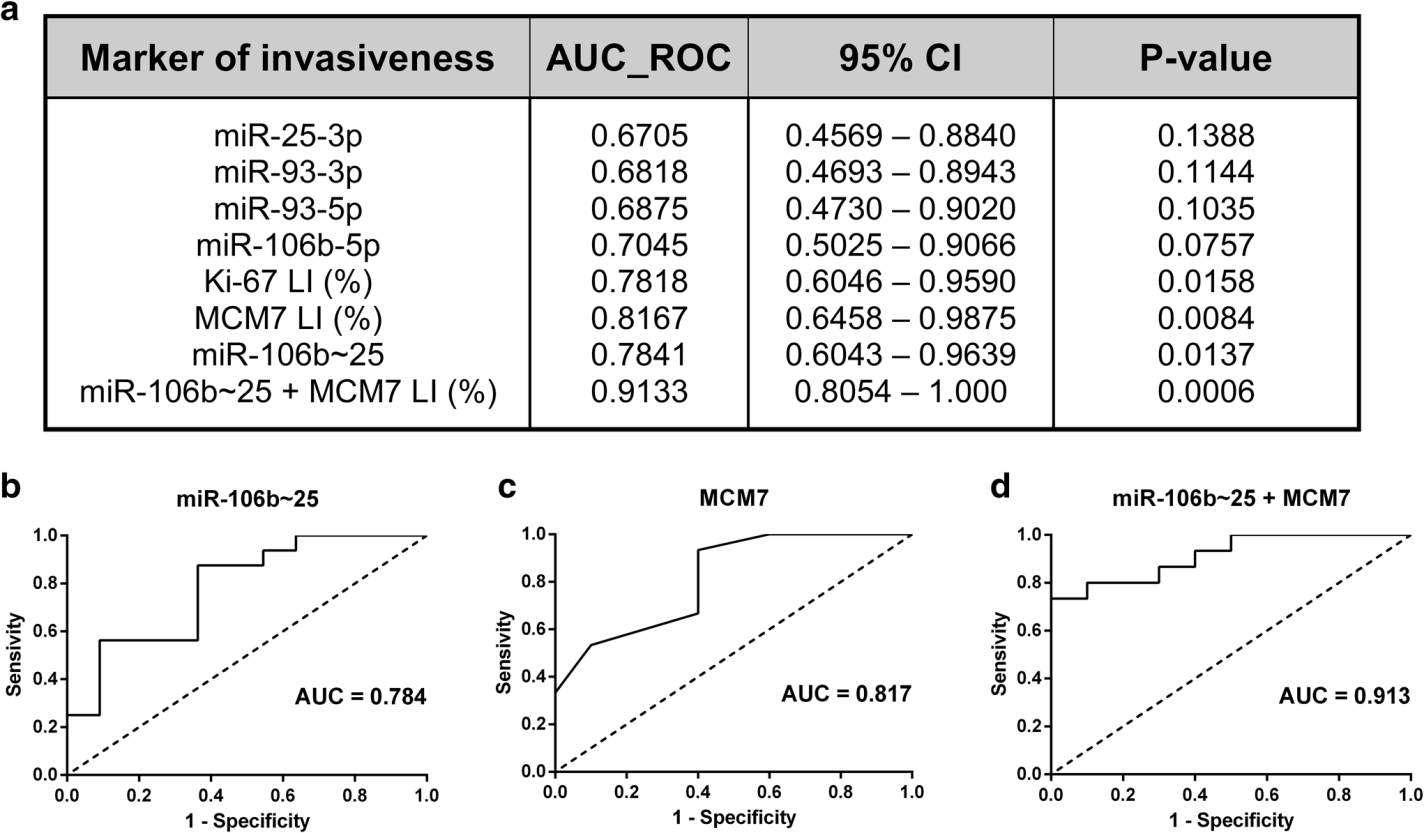
MCM7 LI in combination with the expression of miR-106b~25 is a specific and sensitive test discriminating invasive from noninvasive ACTHomas. **a** In order to establish the role of the IHC biomarkers and microRNAs studied, we analyzed the area under the receiver operating characteristic (AUC ROC). **b** A graphical representation of the three top AUC values

We performed a ROC curve analysis in order to determine the potential usefulness of IHC biomarkers and microRNA expression levels in discriminating invasive from noninvasive PAs (Fig. 7). A multivariate ROC that combined the expression of all four miRNAs from the miR-106b~25 cluster results in a bigger AUC = 0.7841 (95% CI 0.6043–0.9639) than individual miRNAs. Moreover, a combination of the whole miR-106b~25 cluster expression and MCM7 LI distinguished tumor invasiveness even better, as evidenced by AUC = 0.9133 (95% CI 0.8054–1.000).

### High expression of miR-106b~25 and MCM7 is associated with unfavorable surgical outcome and distinguishes recurrent and progressive tumors

**Fig. 8:**
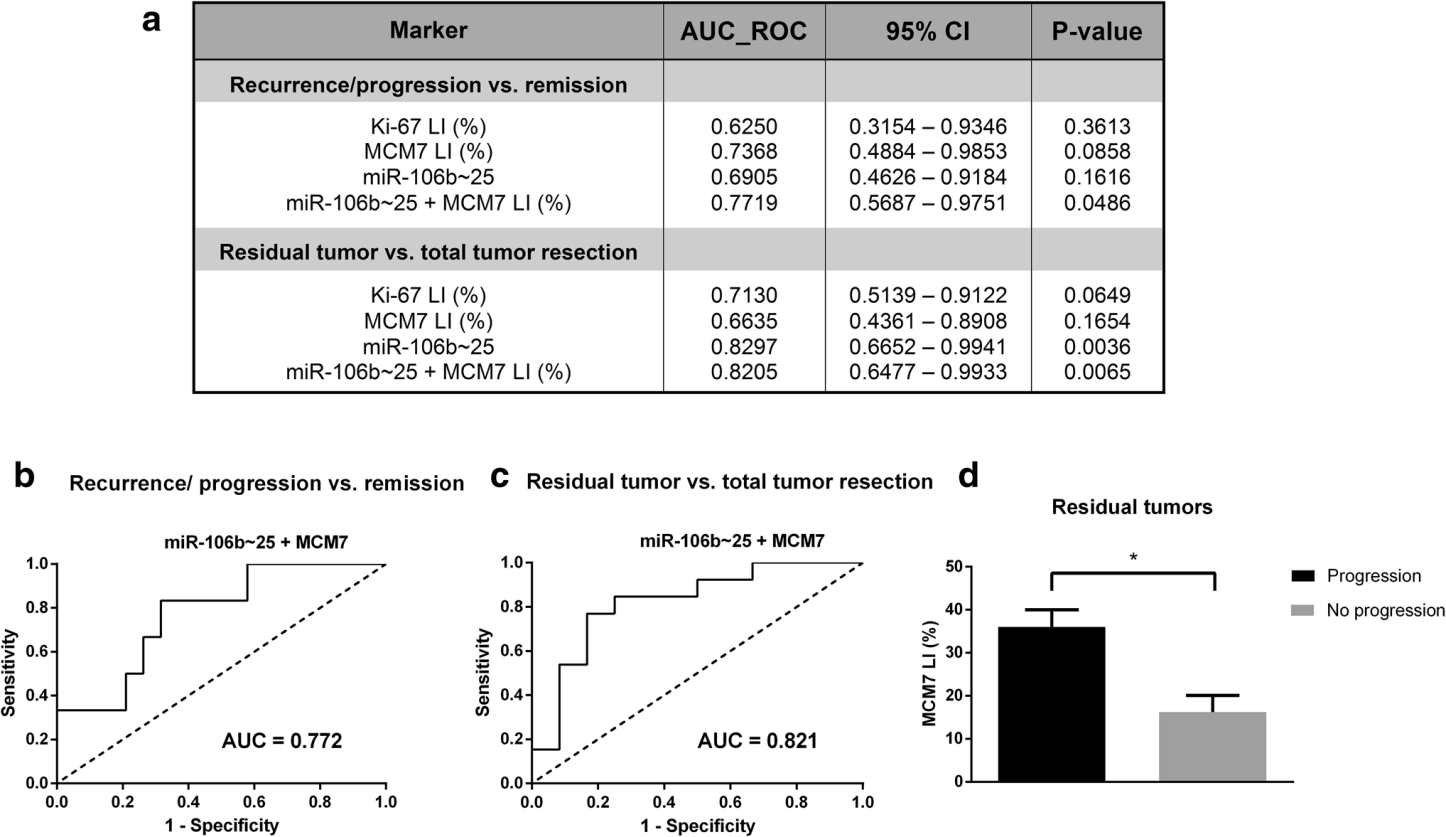
Performance of MCM7 LI, miR-106b~25 expression and Ki67 LI in identifying patients with unfavorable prognosis. **a** A summary of AUC ROC calculated for progression/recurrence and post-operative residual tumor accompanied by a graphical representation of those values (**b**,** c**). **d** The expression of MCM7 in residual tumors that progressed or remained stable calculated using the two-sided Mann–Whitney test: *p < 0.05

The performed surgical procedures resulted in total tumor resection in 12 (48%) out of 25 patients. During the follow-up period (median time: 27 months, range 6–61), recurrence/progression occurred in 6 (24%) out of 25 patients (median time to relapse: 8 months). Because of the limited number of patients in our study, examined biomarkers were not significantly predictive of relapse risk; however they presented a notable discriminatory ability to predict postoperative tumor recurrence or progression as demonstrated by the ROC AUC analysis (Fig. 8a). The combination of miR-106b~25 cluster expression and MCM7 LI was able to accurately (AUC = 0.772; 95% CI 0.5687–0.9751) identify patients with relapse after surgical procedure (Fig. 8b). Furthermore, the expression of miR-106b~25 alone (AUC = 0.830; 95% CI 0.6652–0.9941) and in combination with MCM7 (AUC = 0.821; 95% CI 0.6477–0.9933) discriminated patients with post-surgery residual tumor from those that underwent successful radical operation (Fig. 8a, c). Among residual adenomas, the ones that progressed exhibited significantly higher MCM7 LIs (mean: 36.0 ± 4.0%) than clinically controlled tumors (mean: 16.3 ± 3.9%) (Fig. 8d).

## Discussion

In this study, we demonstrate the expression of the miR-106b~25 cluster and its host gene MCM7 in a group of ACTH-producing PAs and characterize their utility to evaluate tumor invasiveness and clinical behavior. Our results indicate that both miR-106b~25 and MCM7 are overexpressed in a group of invasive tumors. Interestingly, the most prominent overexpression of tested biomarkers was observed in Crooke’s cell adenomas. To our knowledge, this paper’s findings are the first describing miRNA expression in CCAs and co-expression of miR-106b~25 with MCM7 in pituitary tumors.

Over the last decade, accumulating evidence has proven that deregulation of miRNA expression is involved in PAs development and progression [33–35]. In previous studies utilizing microarrays, many miRNAs were shown to be aberrantly expressed in PAs, and the altered expression of some miRNAs has been associated with tumor diameter, invasiveness, and therapeutic outcome [32, 36]. Furthermore, it was demonstrated that each PA subtype can be characterized with a specific miRNA expression profile [37]. Although several miRNAs were demonstrated to be up- or downregulated in ACTH-immunopositive PAs in comparison to normal pituitary tissue [37–41], our current understanding of the biological effects of altered miRNA expression is still limited.

The results of our study are consistent with previous reports demonstrating an important proto-oncogenic role of miR-106b~25 in tumorigenesis and its overexpression in numerous human malignancies [23, 24, 26, 42]. Similarly, an upregulation of miR-106b has been recently described in a study that included 55 human pituitary adenomas with different hormonal activities [42]. Its levels associated with PTEN (phosphatase and tensin homolog) downregulation and the authors suggested that miR-106b-5p targeted PTEN’s mRNA and promoted tumor invasiveness mediated via activation of PI3K/Akt signaling. Another group of authors reported a case of nonfunctioning pituitary carcinoma with overexpressed miR-106b-5p when compared to the primary tumor [43]. Among other miRNAs reported to negatively regulate PTEN expression in pituitary tumors were: miR-17-5p, miR-20a, miR-26b, miR-21 and miR-200c [44–46]. Palumbo et al. [47] found that inhibition of miR-26b and overexpression of miR-128 resulted in the upregulation of PTEN and decreased PI3K-Akt signaling activity, leading to a suppression of the colony formation ability and invasiveness of pituitary tumor cells. In prostate cancer miR-106b-5p, miR-93-5p and miR-25-3p were confirmed as potent regulators of PTEN, and acting in cooperation with MCM7 they were able to initiate prostate oncogenesis [26]. Low PTEN expression and high Akt kinase activity are one of the major oncogenic phenomena driving pituitary neoplasia [48, 49]. These observations, together with the results presented in our study, suggest an involvement of PTEN-targeting miR-106b~25 in the development and progression of pituitary tumors.

Global miRNA profiling of nonfunctioning pituitary adenomas (NFPAs) and normal pituitary has revealed an overexpression of miR-93-5p in tumor tissues [50]. In the same study the authors proposed that the miRNA expression profile observed in NFPAs was associated with inhibition of the TGF-β pathway. Interestingly, miRNAs belonging to the miR-106b~25 cluster were shown to play and important role in the regulation of TGF-β signaling. They were able to inhibit the growth-suppressive functions of this pathway through the repression of downstream mediators p21 and Bim in gastric cancer cells [22]. In another study it has been shown that miR-106b~25 targeted Smad7, activated TGF-β and was responsible for its switch from a suppressive to a pro-tumoral character [51]. In our study we decided to analyze miR-93-3p, a relatively unknown, uncanonical member of miR-106b~25, of an uncertain significance in oncogenesis. It was postulated to be a tumor suppressor [52, 53], however Wang et al. found that it is present in sera of patients with late-stage non-small cell lung cancer and is a predictor of poor survival [54, 55]. Interestingly, the authors underlined a possible interaction of miR-93-3p with Smad7, basing their prediction on bioinformatic analysis.

MCM7, the host gene of the miR-106b~25 cluster, is a member of minichromosome maintenance family of DNA-binding proteins. It forms a heterohexamer that is a key component of the prereplication complex assembly at the replication origin during early G1 phase [56]. MCM proteins are physiologically detected only in replicating cells, and are considered as reliable markers of proliferation. As MCM7 is essential for DNA replication, its abnormally high expression was detected in several different types of cancer [57–59]. High MCM7 protein levels were demonstrated to correlate with prostate cancer progression, poor prognosis of non-small cell lung cancer patients, recurrence of colorectal cancer, as well as distant metastases, high histological grade and poor prognosis is soft tissue sarcomas [58–62]. Examination of MCM7 expression pattern in a series of PAs revealed its significantly higher levels in invasive ACTH-producing adenomas, compared to noninvasive tumors [18]. In the same set of PAs, no significant difference was detected in Ki-67 levels between the invasive and the noninvasive group. Furthermore, high MCM7 levels, but neither TP53 nor Ki-67, were directly associated with risk of recurrence and progression. The findings correspond to the results of our study, in which MCM7 LI was the most accurate IHC biomarker in discrimination between invasive and noninvasive corticotroph tumors.

Similar expression patterns of miR-106b~25 and MCM7 demonstrated in this study suggest that these genes may be regulated in a similar fashion. Their co-transcription occurs during mitotic cell division [63], and is under control of a promoter with multiple binding sites for c-Myc and E2F1 [64, 65], two well-known drivers of pituitary oncogenesis [66, 67]. It is worth noting that the expression levels of MCM7 and its intronic miRNAs are not perfectly correlated. Several studies have recently reported that their uncoupling can be caused by independent transcription of pri-miRNA using an alternative promoter [68, 69], as well as nonsense-mediated decay of one of MCM7 transcript variants [63]. These findings warrant further investigation utilizing mRNA samples, since the protein levels of MCM7 could also be regulated post-transcriptionally [70].

CCAs are a unique subtype of corticotroph adenomas in terms of pathology and clinical course. They are usually invasive, may exhibit aggressive clinical behavior, and often recur with a low success of cure after reoperation and/or radiotherapy [12]. Their incidence is estimated at less than 1% of all pituitary adenomas and up to date only about 80 cases of CCAs have been published in the literature [12, 71]. Although recent ultrastructural and histological findings have made identification of CCAs more conceivable, still little is known about the mechanism underlying the development of these rare adenomas [12]. The higher expression of miR-106b~25 in CCAs compared to other groups of ACTHomas suggests that this cluster of oncogenic miRNAs might contribute to the aggressive behavior of these tumors.

In conclusion, our findings demonstrate for the first time that both MCM7 and miRNAs within the miR-106b~25 cluster (miR-25-3p, miR-93-3p, miR-93-5p and miR-106b-5p) are co-expressed in pituitary tumors and are associated with their invasive character as well as unfavorable outcome after resection. However, due to the limited number of patients included in this study and its retrospective character, further investigations on larger cohorts of patients are needed to validate the diagnostic and prognostic potential of miR-106b~25 and MCM7 in ACTHomas and other pituitary tumor subtypes. A better understanding of miRNA-mediated regulatory networks constitutes a first step toward the identification of reliable biomarkers and development of novel therapeutic strategies, including miRNA-based therapies, for the treatment of pituitary tumors [34, 72].

## References

[1] Melmed S (2011). Pathogenesis of pituitary tumors. Nat Rev Endocrinol.

[2] Cooper O, Melmed S (2012). Subclinical hyperfunctioning pituitary adenomas: the silent tumors. Best Pract Res Clin Endocrinol Metab.

[3] Nieman LK, Ilias I (2005). Evaluation and treatment of Cushing’s syndrome. Am J Med.

[4] Seltzer J, Ashton CE, Scotton TC (2015). Gene and protein expression in pituitary corticotroph adenomas: a systematic review of the literature. Neurosurg Focus.

[5] Syro LV, Rotondo F, Cusimano MD (2015). Current status on histological classification in Cushing’s disease. Pituitary.

[6] George DH, Scheithauer BW, Kovacs K (2003). Crooke’s cell adenoma of the pituitary: an aggressive variant of corticotroph adenoma. Am J Surg Pathol.

[7] Scheithauer BW, Jaap AJ, Horvath E (2000). Clinically silent corticotroph tumors of the pituitary gland. Neurosurgery.

[8] Gomez-hernandez K, Ezzat S, Asa SL, Mete O (2015). Clinical implications of accurate subtyping of pituitary adenomas: perspectives from the treating physician. Turkish J Pathol.

[9] Cooper O, Ben-Shlomo A, Bonert V (2010). Silent corticogonadotroph adenomas: clinical and cellular characteristics and long-term outcomes. Horm Cancer.

[10] Saeger W, Honegger J, Theodoropoulou M (2016). Clinical impact of the current WHO classification of pituitary adenomas. Endocr Pathol.

[11] Felix IA, Horvath E, Kovacs K (1981). Massive Crooke’s hyalinization in corticotroph cell adenomas of the human pituitary: a histological, immunocytological, and electron microscopic study of three cases. Acta Neurochir.

[12] Di Ieva A, Davidson JM, Syro LV (2015). Crooke’s cell tumors of the pituitary. Neurosurgery.

[13] Rotondo F, Cusimano M, Scheithauer BW (2012). Atypical, invasive, recurring Crooke cell adenoma of the pituitary. Hormones.

[14] Raverot G, Jouanneau E, Trouillas J (2014). Management of endocrine disease: clinicopathological classification and molecular markers of pituitary tumours for personalized therapeutic strategies. Eur J Endocrinol.

[15] Lloyd RV, Kovacs K, Young WF, Farrell WE, Asa SL, DeLellis RA, Lloyd RV, Heitz PU, Eng C (2004). Pituitary tumours. World Health Organization classification of tumours: tumours of endocrine organs.

[16] Mete O, Asa SL (2012). Clinicopathological correlations in pituitary adenomas. Brain Pathol.

[17] Mete O, Ezzat S, Asa SL (2012). Biomarkers of aggressive pituitary adenomas. J Mol Endocrinol.

[18] Coli A, Asa SL, Fadda G (2016). Minichromosome maintenance protein 7 as prognostic marker of tumor aggressiveness in pituitary adenoma patients. Eur J Endocrinol.

[19] Di Leva G, Garofalo M, Croce CM (2014). microRNAs in cancer. Annu Rev Pathol.

[20] Lagos-Quintana M, Rauhut R, Lendeckel W, Tuschl T (2001). Identification of novel genes coding for small expressed RNAs. Science.

[21] Bartel DP (2009). MicroRNAs: target recognition and regulatory functions. Cell.

[22] Petrocca F, Visone R, Onelli MR (2008). E2F1-regulated microRNAs impair TGFbeta-dependent cell-cycle arrest and apoptosis in gastric cancer. Cancer Cell.

[23] Li Y, Tan W, Neo TWL (2009). Role of the miR-106b-25 microRNA cluster in hepatocellular carcinoma. Cancer Sci.

[24] Kan T, Sato F, Ito T (2009). The miR-106b-25 polycistron, activated by genomic amplification, functions as an oncogene by suppressing p21 and Bim. Gastroenterology.

[25] Wang H, Liu J, Zong Y (2010). miR-106b aberrantly expressed in a double transgenic mouse model for Alzheimer’s disease targets TGF-β type II receptor. Brain Res.

[26] Poliseno L, Salmena L, Riccardi L (2010). Identification of the miR-106b ~ 25 microRNA cluster as a proto-oncogenic PTEN-targeting intron that cooperates with its host gene MCM7 in transformation. Sci Signal.

[27] Khuu C, Utheim TP, Sehic A (2016). The three paralogous MicroRNA clusters in development and disease, miR-17-92, miR-106a-363, and miR-106b-25. Scientifica.

[28] Di Leva A, Rotondo F, Syro LV (2014). Aggressive pituitary adenomas diagnosis and emerging treatments. Nat Rev Endocrinol.

[29] Knosp E, Steiner E, Kitz K, Matula C (1993). Pituitary adenomas with invasion of the cavernous sinus space: a magnetic resonance imaging classification compared with surgical findings. Neurosurgery.

[30] Righi A, Morandi L, Leonard E (2013). Galectin-3 expression in pituitary adenomas as a marker of aggressive behavior. Hum Pathol.

[31] Ding Y, Wu H, Warden C (2016). Gene expression differences in prostate cancers between young and old men. PLoS Genet.

[32] Livak KJ, Schmittgen TD (2001). Analysis of relative gene expression data using real-time quantitative PCR and the 2(−delta delta C(T)) method. Methods.

[33] Jiang X, Zhang X (2013). The molecular pathogenesis of pituitary adenomas: an update. Endocrinol Metab.

[34] Gentilin E, degli Uberti E, Zatelli MC (2016). MicroRNAs in the pituitary. Best Pract Res Clin Endocrinol Metab.

[35] Wierinckx A, Roche M, Legras-Lachuer C (2017). MicroRNAs in pituitary tumors. Mol Cell Endocrinol.

[36] Bottoni A, Piccin D, Tagliati F (2005). miR-15a and miR-16-1 down-regulation in pituitary adenomas. J Cell Physiol.

[37] Bottoni A, Zatelli MC, Ferracin M (2007). Identification of differentially expressed microRNAs by microarray: a possible role for microRNA genes in pituitary adenomas. J Cell Physiol.

[38] Stilling G, Sun Z, Zhang S (2010). MicroRNA expression in ACTH-producing pituitary tumors: up-regulation of microRNA-122 and -493 in pituitary carcinomas. Endocrine.

[39] Amaral FC, Torres N, Saggioro F (2009). MicroRNAs differentially expressed in ACTH-secreting pituitary tumors. J Clin Endocrinol Metab.

[40] Gentilin E, Tagliati F, Filieri C (2013). miR-26a plays an important role in cell cycle regulation in ACTH-secreting pituitary adenomas by modulating protein kinase Cδ. Endocrinology.

[41] Qian ZR, Asa SL, Siom H (2009). Overexpression of HMGA2 relates to reduction of the let-7 and its relationship to clinicopathological features in pituitary adenomas. Mod Pathol.

[42] Zhou K, Zhang T, Fan Y (2016). MicroRNA-106b promotes pituitary tumor cell proliferation and invasion through PI3K/AKT signaling pathway by targeting PTEN. Tumour Biol.

[43] Wei Z, Zhou C, Liu M (2015). MicroRNA involvement in a metastatic non-functioning pituitary carcinoma. Pituitary.

[44] Liao C, Chen W, Fan X (2013). MicroRNA-200c inhibits apoptosis in pituitary adenoma cells by targeting the PTEN/Akt signaling pathway. Oncol Res.

[45] Shi X, Tao B, He H (2012). MicroRNAs-based network: a novel therapeutic agent in pituitary adenoma. Med Hypotheses.

[46] Chen C-H, Xiao W-W, Jiang X-B (2013). A novel marine drug, SZ-685C, induces apoptosis of MMQ pituitary tumor cells by downregulating miR-200c. Curr Med Chem.

[47] Palumbo T, Faucz FR, Azevedo M (2013). Functional screen analysis reveals miR-26b miR-128 as central regulators of pituitary somatomammotrophic tumor growth through activation of the PTEN-AKT pathway.. Oncogene.

[48] Musat M, Korbonits M, Kola B (2005). Enhanced protein kinase B/Akt signalling in pituitary tumours. Endocr Relat Cancer.

[49] Monsalves E, Juraschka K, Tateno T (2014). The PI3K/AKT/mTOR pathway in the pathophysiology and treatment of pituitary adenomas. Endocr Relat Cancer.

[50] Butz H, Likó I, Czirják S (2011). MicroRNA profile indicates downregulation of the TGFβ pathway in sporadic non-functioning pituitary adenomas. Pituitary.

[51] Smith AL, Iwanaga R, Drasin DJ (2012). The miR-106b-25 cluster targets Smad7, activates TGF-β signaling, and induces EMT and tumor initiating cell characteristics downstream of Six1 in human breast cancer. Oncogene.

[52] Shang X, Li G, Liu H (2016). Comprehensive circular RNA profiling reveals that hsa_circ_0005075, a new circular RNA Biomarker, is involved in hepatocellular carcinoma development. Medicine.

[53] Li G, Qiu Y, Su Z (2013). Genome-wide analyses of radioresistance-associated mirna expression profile in nasopharyngeal carcinoma using next generation deep sequencing. PLoS One.

[54] Wang Y, Lippman SM, Minna JD et al (2012) Pathway-based serum microRNA profiling and late-stage nonsmall cell lung cancer survival. In: Proceedings of the 103rd annual meeting of the American Association for Cancer Research, vol 72. AACR, Chicago, IL. Philadelphia

[55] Wang Y, Gu J, Roth JA (2013). Pathway-based serum microRNA profiling and survival in patients with advanced stage non-small cell lung cancer. Cancer Res.

[56] Tye BK (1999). MCM proteins in DNA replication. Annu Rev Biochem.

[57] Juríková M, Danihel Ľ, Š Polák, Varga I (2016). Ki67, PCNA, and MCM proteins: markers of proliferation in the diagnosis of breast cancer. Acta Histochem.

[58] Ren B, Yu G, Tseng GC (2006). MCM7 amplification and overexpression are associated with prostate cancer progression. Oncogene.

[59] Tamilzhalagan S, Rathinam D, Ganesan K (2017). Amplified 7q21-22 gene MCM7 and its intronic miR-25 suppress COL1A2 associated genes to sustain intestinal gastric cancer features. Mol Carcinog.

[60] Toyokawa G, Masuda K, Daigo Y (2011). Minichromosome maintenance protein 7 is a potential therapeutic target in human cancer and a novel prognostic marker of non-small cell lung cancer. Mol Cancer.

[61] Ishibashi Y, Kinugasa T, Akagi Y (2014). Minichromosome maintenance protein 7 is a risk factor for recurrence in patients with Dukes C colorectal cancer. Anticancer Res.

[62] Hamamoto Y, Shomori K, Nosaka K (2010). Prognostic significance of Minichromosome maintenance protein 7 and Geminin expression in patients with 109 soft tissue sarcomas. Oncol Lett.

[63] Haldar S, Roy A, Banerjee S (2014). Differential regulation of MCM7 and its intronic miRNA cluster miR-106b-25 during megakaryopoiesis induced polyploidy. RNA Biol.

[64] Zhao Z-N, Bai J-X, Zhou Q (2012). TSA suppresses miR-106b-93-25 cluster expression through downregulation of MYC and inhibits proliferation and induces apoptosis in human EMC. PLoS One.

[65] Suzuki S, Adachi A, Hiraiwa A (1998). Cloning and characterization of human MCM7 promoter. Gene.

[66] Fedele M, Pierantoni GM, Visone R, Fusco A (2006). E2F1 activation is responsible for pituitary adenomas induced by HMGA2 gene overexpression. Cell Div.

[67] Pei L (2001). Identification of c-myc as a down-stream target for pituitary tumor-transforming gene. J Biol Chem.

[68] Sikand K, Slane SD, Shukla GC (2009). Intrinsic expression of host genes and intronic miRNAs in prostate carcinoma cells. Cancer Cell Int.

[69] Ramalingam P, Palanichamy JK, Singh A (2014). Biogenesis of intronic miRNAs located in clusters by independent transcription and alternative splicing. RNA.

[70] Chuang C-H, Yang D, Bai G (2012). Post-transcriptional homeostasis and regulation of MCM2-7 in mammalian cells. Nucleic Acids Res.

[71] Takeshita A, Inoshita N, Taguchi M (2009). High incidence of low O6-methylguanine DNA methyltransferase expression in invasive macroadenomas of Cushing’s disease. Eur J Endocrinol.

[72] Krützfeldt J (2016). Strategies to use microRNAs as therapeutic targets. Best Pract Res Clin Endocrinol Metab.

